# Real-world comparison of Rezūm^®^ water vapor therapy and transurethral resection of the prostate using a pragmatic composite definition of procedural success

**DOI:** 10.1007/s00345-026-06315-2

**Published:** 2026-02-25

**Authors:** Teresa Pina-Vaz, Alberto Costa Silva, Margarida Henriques, Hugo Antunes, Pedro Dias, Carlos Martins-Silva, João Alturas Silva, Afonso Morgado

**Affiliations:** 1 Urology Department, University Hospital Center of São João, Alameda Professor Hernâni Monteiro, 4200-319 Porto, Portugal; 2 Faculty of Medicine, University of Porto, Porto, Portugal; 3 Research Network, Faculty of Medicine, University of Porto, Porto, Portugal; 4 Urology Department, Unidade Local da Região de Aveiro, Aveiro, Portugal; 5 Hospital CUF Viseu, Viseu, Portugal

**Keywords:** Benign prostatic hyperplasia, Ejaculation sparing, Minimally invasivetechniques, Prostate enlargement, Transurethral Resection of Prostate

## Abstract

**Purpose:**

To compare Rezūm water vapor therapy and transurethral resection of the prostate (TURP) using a newly proposed composite definition of procedural success that integrates symptom improvement, safety, and preservation of sexual function.

**Methods:**

This multicenter retrospective cohort included 192 matched patients (96 Rezūm, 96 TURP) treated between 2020 and 2024. Groups were matched for age, prostate volume, International Prostate Symptom Score (IPSS), Qmax and post-void residual urine. Procedural success required simultaneous achievement of all the following: ≥50% IPSS reduction, ≥ 50% Qmax improvement, ≥ 50% (or ≥ 1-point) QoL improvement, absence of intraoperative or Clavien–Dindo grade ≥ III complications, preservation of erectile and ejaculatory function, and no retreatment.

**Results:**

Baseline characteristics were comparable between groups. Rezūm was mostly performed as an outpatient procedure (89.6%), while all TURP patients required admission (*p* < 0.001). Catheterization duration was longer after Rezūm (5 vs. 1.5 days, *p* < 0.001). Both techniques significantly improved IPSS, Qmax, PVR, and PSA with no difference in median changes. Ejaculatory preservation favored Rezūm (87.5% vs. 50.0%, *p* < 0.001), whereas retreatment was more frequent (46.9% vs. 15.6%, *p* < 0.001). Overall procedural success was comparable (53.1% vs. 47.9%, *p* = 0.47). Median follow-up was 46 months.

**Conclusions:**

Rezūm and TURP achieved similar overall procedural success. Rezūm offered superior sexual function preservation and outpatient feasibility, whereas TURP provided greater long-term durability, supporting tailored treatment selection.

**Supplementary Information:**

The online version contains supplementary material available at 10.1007/s00345-026-06315-2.

## Introduction

Non-neurogenic male lower urinary tract symptoms (LUTS) encompass a spectrum of voiding and storage disturbances than can be associated with benign prostatic enlargement (BPE) with a major impact on quality of life [1]. A considerable proportion of patients do not achieve satisfactory symptomatic relief with first-line medical therapy or discontinue treatment due to adverse effects. These patients are often hesitant to proceed with surgery, largely because of concerns regarding its potential impact on sexual function [2]. Among the surgical options, transurethral resection of the prostate (TURP) has long been regarded as the gold standard for surgical management of BPE-related LUTS; however, several minimally invasive surgical therapies have emerged, aiming to provide symptom relief while minimizing complications [3].

Water vapor thermal therapy using the Rezūm^®^ system (Boston Scientific, NxThera, Inc., Maple Grove, MN, USA) was approved in 2015 by the FDA and has seen growing adoption [4, 5]. This minimally invasive surgical therapy delivers thermal energy via water vapor, producing targeted cell necrosis in prostatic tissue while sparing surrounding structures such as the peripheral zone, external urinary sphincter, bladder, and rectum from thermal injury [6]. Clinical studies have shown durable improvements in LUTS and quality of life with Rezūm, but most available data derive from a single prospective trial and a few retrospective cohort studies, with limited comparative evidence against other surgical techniques, namely TURP [7, 8]. Guideline recommendations reflect this uncertainty: the American Urological Association (AUA) assigns Rezūm a moderate recommendation with evidence level C, while the European Association of Urology (EAU) has not yet endorsed its use [9, 10].

A recent study introduced a new pragmatic definition of procedural success, proposing a composite threshold that combines symptom improvement, procedural safety, and preservation of sexual function [11].

The aim of this study was to compare perioperative outcomes, short-term adverse events, functional results, and retreatment rates with this new aggregate definition of success between Rezūm and TURP in baseline-matched patients with BPE-related LUTS.

## Methods

The study was approved by the Institutional Review Board (133/23) and conducted in compliance with the Declaration of Helsinki. We conducted a retrospective cohort comparison of men treated with Rezūm or TURP for non-neurogenic male LUTS. All procedures were performed across three centers between January 2020 and December 2024. Clinical records were reviewed to identify eligible Rezūm cases. For the TURP cohort, a larger pool of 673 patients was available; from this, a subgroup of men was matched to ensure comparable baseline characteristics to the Rezūm group.

Patients included in the study had BPE-related LUTS and underwent either Rezūm or bipolar TURP. Only those with prostate volumes between 30 and 80 mL were considered. Patients with prior prostate surgery, history of prostate cancer, or the presence of an indwelling bladder catheter were excluded. Baseline demographics and clinical parameters included age, prostate volume (mL), serum prostate-specific antigen (PSA, ng/mL), International Prostate Symptom Score (IPSS), quality of life (QoL) with QoL IPSS subscale, maximum urinary flow rate (Qmax, mL/s), post-void residual urine volume (PVR, mL), history of erectile dysfunction, and history of anejaculation. Prior and ongoing medical treatments (α-blockers, 5-alpha reductase inhibitors, anticholinergics, and beta-3 agonists) and main motives for surgical intervention were also collected. Short-term adverse events, including the presence of post-operative storage and voiding symptoms, acute urinary retention, gross haematuria, and urinary tract infection (UTI) were assessed during the first postoperative month. Functional and clinical outcomes were assessed with IPSS improvement, Qmax improvement, PVR reduction, PSA reduction, rate of ≥ 5-point IPSS improvement, preservation of ejaculation, and incidence of de novo erectile dysfunction (ED) during, at least, the first postoperative 6-month visits. All patients were reassessed, and Postoperative Patient Global Impression of Improvement (PGI-I) was assessed with the PGI-I Scale and quality of life (QoL) was assessed with QoL IPSS subscale. Clinical records were reviewed to determine the need for retreatment. In addition, any surgical reintervention required for urological complications was recorded.

Procedure success was defined as the achievement of all the following criteria: a ≥ 50% reduction in IPSS, a ≥ 50% improvement in Qmax, a ≥ 50% (or ≥ 1-point) improvement in QoL score, absence of intraoperative complications or postoperative complications including acute urinary retention or Clavien–Dindo grade ≥ III, preservation of both erectile function and antegrade ejaculation and no need for medical/surgical retreatment [11]. A structured follow-up schedule was generally adopted across centers, with routine assessments performed at 1, 3, 6, and 12 months after the procedure. Success was calculated using the latest follow-up data.

### Statistical analysis

Propensity score matching was performed using a logistic regression model incorporating the following baseline variables: age, prostate volume, IPSS, Qmax, and PVR. A 1:1 nearest-neighbor matching algorithm without replacement and a caliper of 0.2 standard deviations of the logit of the propensity score were used. Balance between groups after matching was assessed using standardized mean differences (SMDs), with values < 0.1 indicating adequate balance (Supplementary Table 1). Descriptive statistics were used to summarize patient demographics and baseline characteristics. Paired t-tests and Wilcoxon signed-rank tests were applied to compare pre- and post-treatment outcomes within groups. A p-value of < 0.05 was considered statistically significant.

## Results

A total of 96 patients treated with Rezūm were matched 1:1 to 96 patients treated with TURP, based on age, baseline IPSS, prostate volume, Qmax and PVR. Baseline demographics and clinical characteristics were comparable between groups. Most patients were receiving at least one medical therapy at baseline, with no significant differences in treatment distribution (Table 1).

**Table 1:** Baseline demographic and clinical characteristics of patients treated with Rezūm^®^ water vapor therapy or TURP

	Rezūm *n* = 96	TURP *n* = 96	*p*-value
Age, years - Median (IQR)	62.3 (57.5–66.9)	64.2 (59.1–69.3)	0.280
Prostate volume, cc - Median (IQR)	46.5 (33.0–59.0)	48.3 (35.0–64.0)	0.420
PSA, ng/ml - Median (IQR)	2.2 (1.1–3.1)	2.5 (1.3–3.5)	0.360
IPSS, n (%)			
Mild (0–7)	17 (17.7)	17 (17.7)	
Moderate (IPSS 8–19)	64 (67)	64 (66.7)	
Severe (IPSS > 20)	15 (16)	15 (15.6)	1.000
QoL - Median (IQR)	4.3 (4.0–5.0)	4.5 (4–5)	0.217
Qmax, ml/s - Median (IQR)	10.2 (8.0–13.0)	9.8 (7.0–12.0)	0.400
PVR, mL - Median (IQR)	61.0 (30.0–120.0.0.0)	65.0 (35.0–125.0.0.0)	0.510
History of anejaculation, n(%)	41 (42.3)	36 (37.5)	0.430
Medical treatment, n(%)			1.000
None	1 (1.0)	1 (1.0)	
α-blocker	47 (48.9)	48 (50.0)	
5-alpha reductase inhibitor	48 (50.0)	49 (51.0)	
Anticholinergic	10 (10.4)	11 (11.5)	
Beta 3 agonist	8 (8.3)	7 (7.3)	
Main motive to proceed with intervention			< 0.001
Medication side effects	36 (37.5)	19 (19.8)	
LUTS poorly controlled	25 (26.0)	52 (54.2)	
Discontinue medical therapy	35 (36.5)	25 (26.0)	
Setting, n (%)			< 0.001
Outpatient	86 (89.6)	–	
Admission	10 (10.4)*	96 (100)	
Type of anaesthesia, n (%)			0.160
General/Sedation	90 (93.8)	94 (97.9)	
Spinal	5 (5.2)	2 (2.1)	
Number of injections, Median (IQR)	5 (4–7)	–	–
Median lobe injection, n (%)	32 (33.3)	–	–
Duration of catheterization, days - Median (IQR)	5 (4–7)	1.5 (1–2)	< 0.001
Follow-up time, months - Median (IQR)	44.5 (31.0–66.0)	46.0 (33.0–70.0)	0.550

*IPSS* International Prostate Symptom Score; *IQR* Interquartile Range; *PSA* Prostate-Specific Antigen; *PVR* Post-Void Residual urine volume; *Qmax* Maximum urinary flow rate; *QoL* Quality of life; *TURP* Transurethral Resection of the Prostate; *all discharged the following day

Indications for surgery differed significantly, with medication side effects reported more frequently among Rezūm patients (37.5% vs. 19.4%, *p* < 0.001) and poorly controlled LUTS more frequently among TURP patients (54.8% vs. 26.0%, *p* < 0.001). Rezūm was predominantly performed in the outpatient setting (89.6%), whereas all TURP patients required admission (*p* < 0.001). Median catheterization duration was longer in the Rezūm group (5 (4–7) vs. 1.5 (1–2) days, *p* < 0.001). At 1 month after procedure, storage symptoms and episodes of acute urinary retention were more frequent after Rezūm (*p* < 0.001 and *p* = 0.041, respectively) (Table 2).

**Table 2:** Early postoperative adverse events within 1 month after Rezūm^®^ and TURP procedures

	Rezūm *n* = 96	TURP *n* = 96	*p*-value
Storage symptoms, *n* (%)	30 (31.3)	7 (7.3)	< 0.001
Voiding symptoms, n (%)	10 (10.4)	11 (11.5)	0.640
Gross haematuria, n (%)	3 (3.1)	3 (3.1)	0.940
Urinary tract infection, n (%)	5 (5.2)	4 (4.2)	0.510
Acute urinary retention, n (%)	13 (13.5)	6 (6.3)	0.041
Hospital readmission, n (%)	4 (4.2)	5 (5.2)	0.740

*TURP * Transurethral Resection of the Prostate

Both groups demonstrated improvements in IPSS, Qmax, PVR, and PSA (Table 3). The proportion of patients achieving a ≥ 5-point IPSS improvement was higher in the TURP group (*p* = 0.030). At the last follow-up, retreatment was required more frequently in the Rezūm group compared with TURP (46.9% vs. 15.5%, *p* < 0.001). Medical therapy was restarted in 33.3% of Rezūm patients and 9.7% of TURP patients. Surgical reintervention was also more common after Rezūm (13.5% vs. 5.2%). Optical internal urethrotomy for urethral stenosis was needed in1.3% TURP patients. Early retreatment rates were similar between groups (≤ 12 months: Rezūm 14.6% vs. TURP 12.5%), whereas late retreatments were markedly more frequent after Rezūm (> 12 months: 32.3% vs. 3.1%, *p* < 0.001). Preservation of ejaculation was higher in Rezūm patients (87.5% vs. 50.0%, *p* < 0.001).

**Table 3:** Functional outcomes, sexual function preservation, retreatment rates, and composite procedural success after Rezūm^®^ and TURP

	Rezūm *n* = 96	TURP *n* = 96	*p*-value
IPSS improvement - Median (IQR)	10.0 (7.0–14.0)	12.0 (8.0–16.0)	0.180
≥ 5-point IPSS improvement, n (%)	72 (75.0)	82 (85.4)	0.030
Qmax improvement, ml/s - Median (IQR)	6.0 (4.0–9.0)	7.0 (5.0–9.0)	0.220
PVR reduction - Median (IQR)	32.0 (15.0–60.0)	45.0 (25.0–75.0)	0.120
PSA reduction, ng/ml - Median (IQR)	0.6 (0.3–1.0.3.0)	0.7 (0.4–1.1)	0.210
PGI-I - Median (IQR)	1.5 (1.0–2.0)	1.5 (1.0–2.0)	0.500
Retreatment, n (%)			< 0.001
Medical treatment	45 (46.9)	15 (15.5)	
Surgical treatment	32 (33.3)	9 (9.4)	
TURP	10 (10.0)	3 (3.1)	
Re-Rezūm	1 (1.0)	–	
Enucleation	2 (2.1)	2 (2.1)	
Other procedure*	–	2 (2.1)	
Timing of retreatment			
≤ 12 months, n (%)	14 (14.6)	12 (12.5)	0.670
> 12 months, n (%)	31 (32.3)	3 (3.1)	0.001
Preservation of ejaculation, n (%)	84 (87.5)	48 (50.0)	< 0.001
De novo ED, n (%)	1 (1.0)	2 (2.1)	1.000
Procedure success, n (%)	51 (53.1)	46 (47.9)	0.470
IPSS reduction ≥ 50%, n (%)	70 (72.9)	75 (78.1)	
QoL improvement ≥ 50% (or ≥ 1 point), n (%)	84 (87.5)	82 (85.4)	
Qmax improvement ≥ 50%, n (%)	74 (77.1)	78 (81.3)	
No intra-operative/Clavien ≥ III complications/No AUR, n (%)	83 (86.5)	90 (93.8)	
Preservation of sexual function (erectile function + ejaculation), n (%)	83 (86.5)	46 (47.9)	
No retreatment need (medical/surgical), n (%)	51 (53.1)	81 (84.4)	
Procedure success stratified by timing			
≤ 12 months, n (%)	15 (62.5)	16 (66.7)	0.770
> 12 months, n (%)	51 (53.1)	46 (47.9)	0.320

*AUR* Acute urinary retention; *ED* Erectile Dysfunction; *IPSS* International Prostate Symptom Score; *IQR* Interquartile Range; *NS* = Not Significant; *PGI-I* Postoperative Patient Global Impression of Improvement;* PSA* Prostate-Specific Antigen; *PVR* Post-Void Residual urine volume; *Qmax* Maximum urinary flow rate; *TURP* Transurethral Resection of the Prostate; *Optical internal urethrotomy for urethral stenosis

Considering the composite definition, overall procedural success was comparable between Rezūm and TURP (53.1 vs. 47.9%).

## Discussion

This multicenter retrospective cohort comparison evaluated Rezūm water vapor therapy and TURP) in men with BPE-related LUTS. The two cohorts were baseline-matched, with comparable demographics, prostate volume, PSA, IPSS, Qmax, and PVR. Differences were mainly observed in surgical setting, catheterization duration, and motives for surgery, reflecting the minimally invasive nature of Rezūm and patient selection patterns.

Our results confirm that Rezūm can be safely performed in an outpatient setting, with 89.6% of cases managed without hospitalization, while all TURP patients required admission. This difference is consistent with prior reports emphasizing Rezūm’s ambulatory feasibility and reduced perioperative burden compared with TURP and other transurethral procedures [8].

Motives for surgery differed significantly, with medication side effects reported more frequently among Rezūm patients (37.5% vs. 19.4%) and poorly controlled LUTS more frequently among TURP patients (26.0% vs. 54.8%). This trend indicates that patients opting for Rezūm are often more concerned with adverse effects and ejaculatory preservation, whereas those undergoing TURP are more frequently driven by symptom severity [12].

Catheterization duration was longer after Rezūm (median 5 vs. 1.5 days), in line with the general recommendation of maintaining a catheter following Rezūm to avoid storage symptoms in the immediate postoperative period [4, 13, 14].

Adverse events at one month revealed higher rates of storage symptoms and acute urinary retention after Rezūm, whereas haematuria, urinary tract infection, and voiding symptoms were comparable to TURP group and comparable with other Rezūm series [15]. These findings parallel those from early studies, where storage symptoms and transient retention were common in the initial weeks, generally resolving in the first weeks [4, 14]. Our findings indicate that storage symptoms were more frequent after Rezūm than after TURP, even though α-blockers or anticholinergics were often prescribed empirically in the early postoperative phase for Rezūm, whereas no such medication was routinely used in the TURP group. This difference can be attributed to the local inflammatory response induced by thermal ablation, with subsequent oedema and necrotic tissue sloughing into the prostatic urethra, which transiently aggravates lower urinary tract symptoms [16].

Functional outcomes demonstrated significant improvement in both groups. Median IPSS reduction, Qmax improvement, PVR decrease, and PSA decline were not statistically different between Rezūm and TURP. These results are consistent with previous studies showing IPSS and Qmax improvements with Rezūm [17]. However, the proportion of patients achieving a ≥ 5-point IPSS improvement (recently defined as a minimum level of improvement for a clinical benefit) [18] was higher in the TURP group.

Retreatment rates were higher in the Rezūm group, with 46.9% requiring additional therapy compared with 15.5% after TURP. When considering only surgical retreatment, the rates were 13.5% for Rezūm versus 5.2% for TURP. When retreatments were evaluated over time, both groups demonstrated comparable early retreatment rates within the first postoperative year, suggesting similar short-term procedural efficacy. However, retreatments occurring beyond 12 months were substantially more common after Rezūm, reflecting the possible long-term durability differences between the two techniques. It is also possible that patients selecting Rezūm, who were more often motivated by medication side effects and the desire to preserve sexual function, may have a lower threshold for pursuing additional treatment if symptomatic improvement falls short of expectations. This behavioural pattern could partly contribute to the higher retreatment rate observed in the Rezūm group. These figures are higher than those reported in pivotal Rezūm trials, where the surgical reintervention rate was up to 8% [4, 19, 20]. We included data from a university centre, a district hospital, and private practice, thereby reflecting real-life practice. This difference may be explained by the longer follow-up in our study, differences in patient selection, and heterogeneity of surgical practice across multiple centres. Although the handpiece can deliver up to 15 injections, the number should be adjusted proportionally to prostate size. Given our cohort’s median prostate volume of 46.5 mL, our median of 5 injections is consistent with standard Rezūm technique [21].

A considerable proportion of patients in both groups presented with anejaculation prior to surgery, most likely associated with previous medical therapy. Preservation of ejaculation was significantly greater in Rezūm patients (87.5% vs. 50.0%), consistent with data showing maintenance of sexual function in most men treated with Rezūm. This remains a key advantage over TURP, where anejaculation occurs in 50–70% of patients [22]. Our rate of ejaculatory preservation after Rezūm was lower than typically reported (~ 97%) [11]. It is important to note that this figure does not reflect the technical performance of the procedure alone, but rather the combined effect of the technique itself, suboptimal symptom control in some patients, and the subsequent need for additional medical therapy which can influence that outcome. De novo erectile dysfunction was uncommon in both groups, with no significant difference observed between Rezūm and TURP. These findings are consistent with previously published long-term data indicating that Rezūm has minimal impact on erectile function [11, 12]. Notably, the incidence of erectile dysfunction in our TURP group was lower than the rates reported in the literature, which range from 3.4% to 32% [23].

A recent study proposed a broader and more pragmatic definition of procedural success, incorporating symptom improvement, urinary flow parameters, quality of life, sexual function preservation, complications, and the need for retreatment into a single composite outcome [11]. According to this definition, a procedure is considered successful only when all criteria are simultaneously fulfilled. Consequently, the overall success rate is inherently determined by the domain with the poorest result, which can disproportionately penalize certain techniques. Using this definition, overall procedural success was comparable between Rezūm and TURP (53.1% vs. 47.9%, *p* = 0.470). Although these are not exceptionally high rates, they reflect a balanced and realistic view of long-term treatment efficacy. Rezūm was primarily penalized by its higher retreatment rate; however, many patients willingly accept this trade-off, preferring to preserve sexual function and quality of life before committing to a definitive surgical procedure [24]. Conversely, TURP outcomes were primarily limited by lower rates of ejaculatory preservation. This should not be interpreted as a procedural failure but rather as an expected consequence of the technique, which remains an effective surgical option for selected patients. It is important to acknowledge that treatment success is not static. Outcomes may fluctuate over time depending on follow-up duration, disease progression, and patient expectations, emphasizing the need for long-term and individualized assessment of therapeutic efficacy.

The relatively long median follow-up of 46 months strengthens the validity of our findings, as it allows for a more robust assessment of treatment durability and retreatment rates compared with most published series. To our knowledge, only two Rezūm studies have reported comparable long-term outcomes, with follow-up durations longer than 20 months [4, 16].

Limitations of our study include the retrospective design, lack of standardized postoperative prostate volume measurements, and possible underestimation of early storage symptoms given the frequent use of α-blockers or anticholinergics postoperatively. A cost analysis was not feasible, as the study encompassed institutions operating under different administrative frameworks. Prostates larger than 80 g and chronically catheterized patients were not included in this study, which limits the applicability of our findings to these common subpopulations. A further limitation is that erectile function and ejaculatory outcomes were not evaluated using validated questionnaires. Additionally, although both cohorts were baseline-matched, residual confounding cannot be excluded given the retrospective design.

Our study adds meaningful real-world evidence by providing multicentre comparative data on Rezūm and TURP in baseline-matched patients with prostates of 30–80 mL. Both procedures resulted in significant improvements in LUTS and urinary flow, while demonstrating distinct procedural profiles. Rezūm offered outpatient feasibility and better preservation of ejaculatory function but was associated with a longer catheterisation period, more early postoperative storage symptoms, and a higher retreatment rate, particularly over the medium to long term. Within the constraints of the composite definition, the overall success rates appeared similar; however, the domains driving limitations differed between techniques. Rezūm was mainly affected by retreatment, whereas TURP was limited by ejaculatory dysfunction. As these components hold different importance for individual patients, the numerical similarity in overall success should not be interpreted as equivalence across clinically meaningful dimensions. Instead, these findings highlight the inherent trade-offs of each approach and reinforce the importance of patient-centred counselling that aligns treatment choice with personal priorities regarding durability, sexual function, and tolerance for potential retreatment.

While retrospective and real-world data provide valuable insights into Rezūm outcomes, randomized controlled trials directly comparing Rezūm with other surgical techniques are still lacking. Such trials are necessary to better define the relative efficacy, durability, and safety profile of Rezūm within the surgical treatment landscape for BPE.

## Supplementary Information

Below is the link to the electronic supplementary material.

Supplementary Material 1

## Data Availability

The data that support the findings of this study are restricted by local ethics comissions. The data are, however, available from the authors upon reasonable request and with the permission of ethics comitee.
