# Supplementary material for: Real-world comparison of Rezūm® water vapor therapy and transurethral resection of the prostate using a pragmatic composite definition of procedural success
Source: World J Urol. 2026 Feb 25;44(1):196. doi: 10.1007/s00345-026-06315-2 (PMC12935790; doi:10.1007/s00345-026-06315-2)
Supplement: Supplementary file 1 — Supplementary Material 1 [file 345_2026_6315_MOESM1_ESM.docx]

Supplementalary table 1. Propensity score matching

| **Variables** | **SMD before matching** | **SMD after matching** |
| --- | --- | --- |
| Age | 0.21 | 0.04 |
| Prostate volume | 0.18 | 0.03 |
| IPSS | 0.25 | 0.07 |
| Qmax | 0.17 | 0.05 |
| PVR | 0.12 | 0.02 |
